# Effectiveness of a family-centered behavioral and educational counselling approach to improve periodontal health of pregnant women: a randomized controlled trial

**DOI:** 10.1186/s12903-020-01265-6

**Published:** 2020-10-16

**Authors:** Pei Liu, Weiye Wen, Ka Fung Yu, Xiaoli Gao, Edward Chin Man Lo, May Chun Mei Wong

**Affiliations:** 1 Dental Public Health, Faculty of Dentistry, The University of Hong Kong, Pokfulam, Hong Kong, SAR China; 2 Department of Stomatology, Beijing Friendship Hospital, Capital Medical University, Xicheng District, Beijing, China; 3 Faculty of Dentistry, National University of Singapore, Singapore, Singapore; 4 Saw Swee Hock School of Public Health, National University of Singapore, Singapore, Singapore

**Keywords:** Oral health promotion, Behavioral intervention, Pregnant women, Periodontal disease, Oral hygiene

## Abstract

**Background:**

Poor oral hygiene and high hormone levels during pregnancy can lead to a deterioration in periodontal health. This study assessed the effectiveness of a family-centered behavioral and educational counselling program on improving the periodontal health of women during pregnancy and postpartum.

**Methods:**

A randomized controlled trial was conducted among pregnant women (10th-22nd gestational week) and their husbands. Participating families were randomized into test and control groups. Intervention in the test group included explanation of oral health education (OHE) pamphlets, oral hygiene instruction, individualized feedback, and proposed solutions to overcome barriers in self-care. Reinforcements were implemented in the 3rd trimester of pregnancy and six months postpartum. In the control group, only OHE pamphlets were distributed. The assessed outcomes were bleeding on probing (BOP), periodontal pocket (Poc), loss of clinical attachment (LoA), and Visible Plaque Index (VPI). The data collection was carried out at baseline (T_0_), in the 32nd gestational week (T_1_), and 12 months postpartum (T_2_).

**Results:**

Altogether 589 pregnant women were recruited, and 369 attended all three visits (test:188; control:181). In the test group, the mean VPI score at T_0_ was 0.19, which decreased to 0.14 at T_1_ and 0.15 at T_2_. In the control group, the mean VPI decreased from 0.19 at T_0_ to 0.16 at T_1_, but increased to 0.22 at T_2_. A main effect of time and intervention and an interaction between time and intervention were detected (all *p* < 0.05), indicating that the intervention effect differed between T_1_ and T_2_. The test group showed a significantly greater decrease over time than the control group did. Similarly, the mean BOP% decreased more significantly over time in the test group (T_0_:57%, T_1_:46%, T_2_:35%) than in the control group (T_0_:58%, T_1_:52%, T_2_:46%). For Poc and LoA, there were improvements in both study groups at 12 months postpartum, compared with during pregnancy (*p* < 0.001).

**Conclusions:**

Providing family-centered, behavioral, and educational counselling to pregnant women at an early stage of pregnancy and with reinforcements can improve their oral hygiene and reduce gingival inflammation. The effect can be sustained over an extended period and is greater than that of distributing oral health leaflets alone.

**Trial registration:**

Clinicaltrials.gov, #NCT02937194. Registered 18 October 2016. Retrospectively registered, https://clinicaltrials.gov/ct2/show/NCT02937194?cond=Family-centered+oral+health+promotion+for+new+parents+and+their+infants&draw=2&rank=1

## Introduction

Maintaining good oral health is essential for a healthy pregnancy. Pregnancy-induced hormonal changes can affect periodontal tissues. Elevated hormone levels during pregnancy amplify the plaque-induced gingival inflammatory response, resulting in swelling and bleeding [1–3]. The prevalence of pregnancy gingivitis has been reported to be 36–100% [4–7]. The severity of gingival inflammation usually increases from the 16th to the 40th week of pregnancy and decreases after parturition [8, 9]. Some studies have reported increases in periodontal pocket (Poc) depth or loss of attachment (LoA) during pregnancy [3, 8, 10]. However, the differences in study design (e.g., variability of pre-existing periodontal status at baseline, lack of a non-pregnant comparison group, or a short follow-up period) make it difficult to conclude if pregnancy exacerbates periodontal damage [11].

Periodontal diseases are initiated by dental plaque, and untreated gingivitis can lead to further insult of the periodontal tissues. Periodontal disease can be prevented through oral health education (OHE). Educating pregnant women to perform effective plaque removal is imperative for the prevention and control of periodontal disease in this vulnerable group. Despite this, a recent systematic review reported that, in most of the studies, oral health promotion among pregnant women mainly involved delivering messages on infant oral health, instead of focusing on pregnant women’s oral health [12]. Some studies that targeted pregnant women only reported their oral health knowledge, oral hygiene practices, or caries status [13–16]. Three studies reported the periodontal health outcomes, among which two studies delivered periodontal treatments to pregnant women who already had gingivitis, and the other was a short-term study (4 weeks) [17–19]. No evidence-based conclusions could be drawn from their findings. The effectiveness of OHE for pregnant women remains a research gap.

Traditionally, there has been an emphasis on OHE for individuals. Current concepts of OHE acknowledge the importance of involvement at the individual, family, and community levels [20]. Studies have also reported the interrelationships between oral health practices and status among married couples, parents, and their children [21, 22]. Thus, delivering OHE to members in the family together (a family-centered approach) is likely to be more effective in improving the oral health of family members than delivering conventional OHE to individuals. The aim of this study was to evaluate the effectiveness of a family-centered behavioral and educational counselling approach in improving the periodontal health of women during pregnancy and 12 months postpartum, as compared with traditional OHE (distributing pamphlets alone). It was hypothesized that this approach would improve the periodontal health of pregnant women.

## Materials and methods

This study was part of a randomized controlled trial aimed to decrease the incidence of early childhood caries (ECC). A family-centered approach was used to provide behavioral and educational counselling to pregnant women and their husbands to establish self-efficacy of the new parents in their oral health self-care and oral health care for their infants so as to decrease ECC in their children at 3 years old. Comprehensive data of the pregnant women, their husbands, and their babies were collected and analyzed in the 10th-22nd gestational weeks (T_0_), in the 3rd trimester of pregnancy (T_1_), and when the baby was 1 year old (T_2_), 2 years old (T_3_), and 3 year**s** old (T_4_). The study protocol was approved by the Institutional Review Board of the University of Hong Kong (#UW 13–163) and the Research Ethics Boards at each of the participant recruitment sites, as well as registered on ClinicalTrials.gov (#NCT02937194).

### Study population and participant recruitment

The pregnant women and their husbands were recruited from the obstetrics and gynecology department of three public hospitals and from two maternal and child health centers in Hong Kong. The potential participants were approached during their prenatal visits with minimal disturbance to the routine care at the recruitment sites. The inclusion criteria were women 1) with first-time pregnancy; 2) between 10 and 22 weeks of gestation; 3) 18 years or older at enrollment; and 4) who could understand written and spoken Cantonese. Pregnant women were excluded if they were 1) not of Chinese ethnicity or 2) had a severe systemic disease. Written informed consent was obtained from both the pregnant woman and the husband. The recruitment period was from June 2014 to June 2016.

### Randomization and blinding

This was a randomized controlled trial with parallel design (1:1 allocation ratio). The participants were randomly assigned to the test and control groups after collection of the baseline data, which included a self-completed questionnaire and dental examination conducted by dentists who were not involved in the intervention. An independent statistician generated the random number sequence in Excel before recruitment. Block randomization with a block size of 4 was adopted. Opaque sealed envelopes were used to conceal the allocations. The research assistants, who enrolled the participants in this study and collected the questionnaire information, and the dental examiners were blinded to the participants’ group allocation.

### Intervention

The intervention provided to the pregnant women and their husbands in the test group was family-centered behavioral and educational counselling, which occurred in the early stages of pregnancy (T_0_) and was further reinforced in the late stage of pregnancy (T_1_) and 6 months after delivery. The process of the intervention was interactive and supported participants to progress toward their goals, i.e., being healthy for both themselves and their babies. They received specific, action-oriented advice, rather than general information on oral health behaviors, to achieve the goals.

At T_0_, a 20- to 40-min individualized counselling session was given by a trained dental auxiliary staff member to both the expectant mother and her spouse. The whole process was as follows:

1. Face-to-face explanation of two OHE pamphlets: Each family was given two OHE pamphlets produced by the Department of Health of the Hong Kong SAR government. The first OHE pamphlet entitled “*Cleaning your teeth by toothbrushing*” was intended for the general public and included information on i) the etiology and pathological progress of dental caries and periodontal disease; ii) description of plaque, tooth anatomy, and how oral bacteria cause oral diseases; iii) recommended oral health self-care measures, including brushing teeth at least twice daily using fluoridated toothpaste and daily flossing; and iv) illustrations on toothbrushing and flossing. The second OHE pamphlet entitled “*Oral health for the expectant mother*” introduced the changes in oral health during pregnancy, the potential link between periodontitis and adverse birth outcomes, and the appropriate time for a dental visit during pregnancy. The benefits of toothbrushing and flossing, as well as the impacts of poor oral hygiene during pregnancy (e.g., susceptibility to periodontal diseases, increased risk of preterm delivery, and increased risk of ECC through vertical transmission of oral bacteria) were emphasized.

2) Demonstration of toothbrushing and dental flossing: Manual toothbrushing technique (Bass method as recommended by the American Dental Association) was demonstrated on a tooth model. The *“tell-show-do”* technique was used with the participants listening to the explanation, observing the proper technique for toothbrushing and flossing, and practicing on the models.

3) Proposing possible ways to overcome barriers related to toothbrushing and flossing during pregnancy: The participants were encouraged to voice their perceived barriers in performing toothbrushing and flossing, and advice on overcoming these barriers were provided. For example, it was suggested that the pregnant women use a soft, small-head toothbrush to reduce the risk of nausea or vomiting; concentrate on breathing when cleaning the posterior teeth; and brush without toothpaste but rub the teeth with a small amount of fluoride toothpaste after brushing.

4) Providing individualized advice based on the dental checkup findings and answering the questions raised by participants: Questions raised by participants included, *“Is it safe to use mouthwash during pregnancy?” “Is an electric toothbrush better than a manual toothbrush?”* and *“Should I switch to fluoride-free toothpaste to decrease the potential harm to my fetus?”*

At the 3rd trimester (around the 32nd gestational week) (T_1_), the expectant mothers and their husbands self-reported their performance in toothbrushing and flossing. The instructions on oral hygiene practices were reinforced if they disagreed with any of the self-efficacy statements: “*I am confident that I can brush my teeth twice per day*”; “*I am confident that I can brush my teeth and use the dental floss correctly*”; “*I am confident that I can clean my oral cavity well*.”

After the babies were born and reached 6 months old, mothers in the test group were contacted through telephone calls or messages. The instructions on proper oral hygiene practice for themselves and their babies were reinforced. In addition, encouragement and information on performance assessment and barrier identification were provided to the participants.

The only intervention in the control group was the delivery of OHE pamphlets for adults and pregnant women. There was no further reinforcement after baseline data collection.

### Sample size calculation

As mentioned earlier, this study was part of a clinical trial that aimed to reduce the incidence of ECC. Sample size calculation for this trial was based on the anticipated rate of ECC among children at 3 years old, and the result showed that a total of 584 families were required. Even allowing for a 40% dropout rate, i.e., with 350 women (175 in each group), there would still be at least 88% power to detect an absolute difference of 0.05 (or 5%) in the mean Visible Plaque Index (VPI) score (assuming SD = 0.15) or percentage of sites with bleeding on probing (BOP%) between the test and control groups at a statistical significance level of 0.05. Thus, the sample size calculation based on ECC outcome was sufficient for detecting changes in the periodontal health parameters of the pregnant women.

### Data collection

Data were collected from the participants via dental examination and a questionnaire at T_0_, T_1_, and T_2_. Oral hygiene status was recorded using the VPI, which reflects the overall level of plaque accumulation [23]. The presence or absence of visible plaque on the buccal and lingual surfaces of six index teeth (tooth 16, 21, 24, 36, 41, 44) was recorded. Periodontal status was measured using the periodontal examination methods and indices recommended by the World Health Organization (WHO) [24]. A CPI probe with a 0.5-mm ball-ended tip and scale marked at 3.5/5.5 mm was used to probe the WHO recommended index teeth (one tooth in each sextant) [24]. For each tooth, the highest code that corresponded with the most severe condition among six probing sites was recorded. One score represents one sextant. Gingival inflammation was detected by BOP. The presence or absence of bleeding after gentle probing of periodontal sites was recorded. Poc depth, measured from the free gingival margin to the base of the periodontal pocket, and LoA, measured from the cemento-enamel junction to the base of the periodontal pocket, were recorded to the nearest 3.5- or 5.5-mm mark of the CPI probe.

The baseline and follow-up examinations of oral hygiene and periodontal status were conducted by two calibrated examiners, who were trained by an experienced epidemiologist prior to the data collection. At each time point, duplicate examinations were conducted on 5% of the participants. The inter-examiner reproducibility was good (VPI: Kappa = 0.77, BOP: Kappa = 0.76, Poc: Kappa = 0.80, LoA: Kappa = 0.80). The intra-examiner reproducibility was good for both examiners (all Kappa > 0.75).

Before each dental examination, the participants completed a questionnaire, which included three questions on oral hygiene practices (toothbrushing frequency, use of fluoridated toothpaste, and additional oral hygiene measures) and twelve questions on knowledge of periodontal health. Information on the participants’ sociodemographic background and smoking and alcohol consumption habits was also collected.

In the present study, the primary outcome of the interventions was periodontal health. The BOP%, the number of sextants with Poc > 3 mm (NPoc), and the number of sextants with LoA > 3 mm (NLoA) were the primary outcome variables. The secondary outcome was oral hygiene status measured by the percentage of examined tooth surfaces with visible plaque (VPI score). A participant whose VPI score = 0, BOP% = 0, NPoc = 0, and NLoA = 0 was defined as having healthy periodontal condition [24].

### Statistical analysis

Statistical analysis was performed using SPSS Statistics for Windows (IBM, Version 24.0. Armonk, NY: IBM Corp). Data of the participants who attended all three examinations were included in the analysis. To compare the characteristics between the test and control groups and between participants who completed the follow-ups and those who did not, Mann-Whitney U tests and χ^2^ tests were performed to assess the differences in the medians of the continuous variables and the distribution of categorical variables between groups, respectively.

The effects of the intervention and time of examination on the BOP% and the VPI score (continuous variables, 0–100%) were analyzed using a mixed-effect ANOVA model. Because the frequency distribution of the NPoc and NLoA was highly skewed with excessive zero scores (negative binomial distribution), a generalized linear mixed model (GLMM) on count data was adopted. In the above models, time point (T_0_, T_1_, and T_2_) was regarded as the within-subjects factor, and the intervention group allocation was the between-subjects factor. The interaction between time point and group allocation tested whether the intervention effect differed between T_1_ and T_2_. The participants’ age, gestational month, education level, monthly income, dental scheme coverage, and smoking habit were input into the initial model as confounding factors. Insignificant variables were removed. The *p*-values of the pairwise comparisons were adjusted by Bonferroni adjustment, and the level of statistical significance used in all tests was 0.05.

## Results

**Fig. 1:**
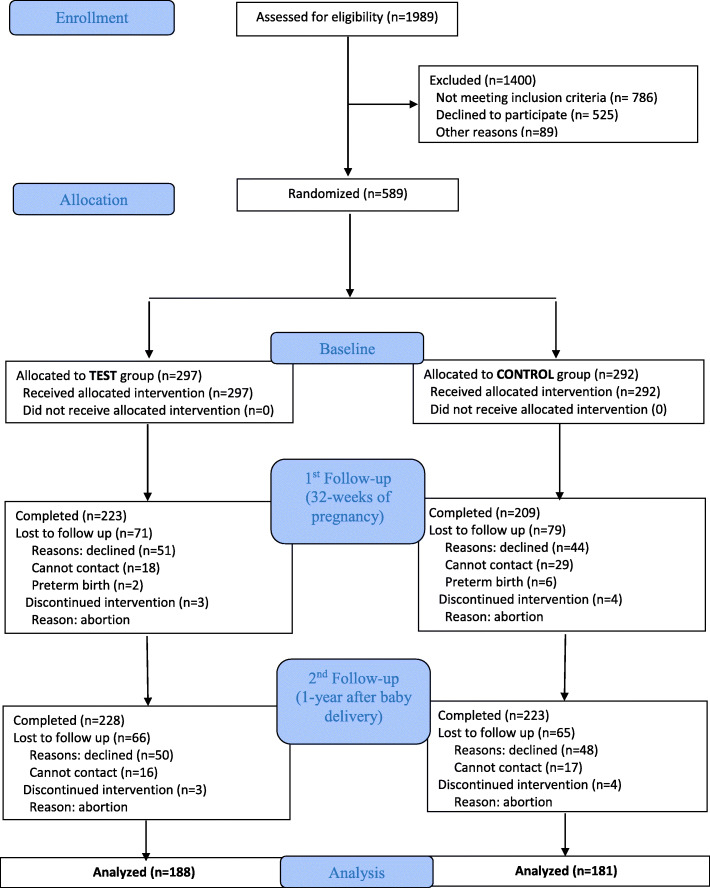
CONSORT diagram of participants on recruitment, randomization and follow-ups. The number of participants of each follow-up was compared to the baseline. The follow-up rate of each time was more than 75%. The reasons for loss of follow-up were participant refusal, transferring to other health centers, hospitals for later antenatal checkups which were different from the ones included in this study, loss of contact, preterm delivery before 32nd gestational week or abortion. The data analysis was based on the participants who completed both the 1st and 2nd follow-ups

Figure 1 shows the CONSORT flowchart for the recruitment, randomization, and follow-ups of the participants. A total of 1989 pregnant women were approached, of whom 1203 met the eligibility criteria, and 589 participants with complete baseline data were recruited for this study (participation rate 49.0%). Through the random allocation, 297 and 292 participants were assigned to the test and control groups, respectively.

Table 1 summarizes the characteristics of the pregnant women who participated in the trial at baseline. Their mean age was 31.1 years (SD = 4.0), and the mean gestational age at the time of recruitment was 14.4 weeks (SD = 2.6). More than 60% of the participants had an income of HK$30,000 or more per month, which is above the average level of Hong Kong households. More than two thirds (67.1%) of the participants had received tertiary education. Less than half (40.4%) of the participants were covered by a dental care scheme. There were no statistically significant differences in the demographic characteristics, dental scheme coverage, and alcohol drinking habit between the test and control groups (*p* > 0.05). In contrast, there was a higher proportion of participants with a smoking habit in the control group than in the test group (*p* = 0.014).

**Table 1:** Socio-demographic backgrounds and other characteristics of the participants at baseline

	Control group (*n* = 292)	Test group (*n* = 297)	Total (*n* = 589)	*p*-value*
**Age** (years, mean ± SD)	31.1 ± 4.2	31.2 ± 3.8	31.1 ± 4.0	0.566
**Gestational age** (weeks, mean ± SD)	14.5 ± 4.2	14.3 ± 2.6	14.4 ± 2.6	0.491
**Education Level** (%)				0.094
Up to junior high school	8.6	11.1	9.8	
Senior high school	26.7	19.5	23.1	
Tertiary education	64.7	69.4	67.1	
**Monthly household income** (%) **				0.204
HK$19,999 or less	18.6	15.2	16.9	
HK$20,000-29,999	21.6	19.6	20.6	
HK$30,000-39,999	15.8	21.3	18.6	
HK$40,000-59,999	25.4	29.4	27.4	
HK$60,000 or more	18.6	14.5	16.5	
**Dental scheme coverage** (%)				0.611
No	61.6	59.6	60.6	
Yes	38.4	40.4	40.4	
**Smoking prior to pregnancy** (%)				0.014
No	93.7	98.2	96.0	
Yes	6.3	1.8	4.0	
**Alcohol use prior to pregnancy** (%)				0.322
No	81.6	85.1	83.4	
Yes	18.4	14.9	16.6	
**VPI** (mean ± SD)(0 ~ 1)	0.20 ± 0.16	0.19 ± 0.16	0.20 ± 0.16	0.588
> 0.5 (%)	6.8	7.2	6.6	
**BOP %** (mean ± SD)(0 ~ 100%)	59 ± 27	58 ± 26	58 ± 27	0.847
> 50 (%)	74.7	71.4	73.0	
**Pocket** (%)				0.774
Absence	89.0	87.9	88.4	
4-5 mm	9.6	11.1	10.4	
6 mm or more	1.4	1.0	1.2	

* p-value obtained using Mann-Whitney U test for continuous data and X^2^ test for categorical data, the comparison of the distribution of smoking was tested by X^2^ exact test

** The monthly household income information was missing for two enrolled participants

At baseline, although visible plaque was detected in most (82.5%) of the study participants, only 6.6% of them had visible plaque on more than 50% of the examined tooth surfaces. Mean percentage of tooth surfaces with plaque were similar in the test and control groups at baseline (*p* > 0.05). Almost all (95.2%) of the participants had BOP. Over 70% of the participants had BOP in more than 50% of the periodontal probing sites. Nearly 90% of the participants were free of Poc in the sextants examined. There was no statistically significant difference between the two groups regarding the percentage of sites with BOP and the number of sextants with Poc (*p* > 0.05).

Table 2 shows the characteristics of participants who were followed up and those who were not. Of the 589 participants recruited, 369 (62.6%) attended both the T_1_ and T_2_ follow-up examinations and completed the data collection. Participants who were lost to follow-up were younger (*p* < 0.01). There was no statistically significant difference (*p* > 0.05) in other characteristics between the participants who completed the follow-ups and those who did not.

**Table 2:** Comparison of the characteristics of participants with completed data vs. without completed data

	Followed-up participants				Drop-out participants				Followed-up vs. Drop-out *p*-value*
	Test (*n* = 186)	Control (*n* = 183)	Total (*n* = 369)	*p*-value*	Test (*n* = 110)	Control (n = 110)	Total (*n* = 220)	*p*-value*	
**Age** (year, mean ± SD)	31.2 ± 3.7	31.9 ± 4.2	31.5 ± 4.0	0.765	31.0 ± 3.8	30.5 ± 4.1	30.4 ± 4.4	0.449	< 0.01
**Gestational age** (weeks, mean ± SD)	14.3 ± 2.7	14.5 ± 2.9	14.4 ± 2.7	0.439	14.3 ± 2.5	14.3 ± 2.5	14.3 ± 2.7	0.983	0.644
**Education level (%)**									
Junior high school or below	9.4	7.6	9.0	0.633	15.5	11.0	10.9	0.043	0.063
Senior high school	19.2	22.4	20.4		20.2	37.8	27.7		
Tertiary education or above	71.4	70.0	70.6		64.3	51.2	61.4		
**Dental scheme coverage** (%)									
No	57.3	59.5	61.2	0.639	67.9	68.3	63.0	0.952	0.669
Yes	42.7	40.5	38.8		37.0	31.7	37.0		
**Monthly household income** (HKD, %)									
< $20,000	13.1	15.7	14.7	0.205	20.2	25.6	20.1	0.438	0.320
$20,000- < $30,000	18.3	18.1	19.9		22.6	31.7	21.9		
$30,000- < $40,000	22.1	16.2	20.4		20.2	14.6	15.5		
$40,000- < $60,000	31.0	27.1	28.3		25.0	20.7	26.0		
> $60,000	15.5	22.9	16.6		11.9	7.3	16.4		
**Smoking** (%)									
No	95.8	96.3	96.2	0.369	94.8	95.2	95.1	0.688	0.650
Yes	4.2	3.7	3.8		5.2	4.8	4.9		

* p-value obtained using Mann-Whitney U test for continuous data and X^2^ test for categorical data, X^2^ exact test for smoking

Table 3 presents the VPI score, BOP%, NPoc, and NLoA of the test and control groups and the between-group differences at the three time points. In the mixed-effect ANOVA models on VPI% and BOP% and GLMM on NPoc and NLoA, the participants’ age, gestational month, education level, monthly income, dental scheme coverage, and smoking habit were not statistically significant (all *p* > 0.05). These variables were removed in the final models.

**Table 3:** Distribution of periodontal outcomes and comparison of change between groups over time

	Test Group (*n* = 188)			Control Group (*n* = 181)			#*p*-value		
	Baseline (T_0_)	1st Follow-up (T_1_)	2nd Follow-up (T_2_)	Baseline (T_0_)	1st Follow-up (T_1_)	2nd Follow-up (T_2_)	Time	Time *Group	Group
**Healthy** **Perio-conditions** (n, %)	4 (2.2%)	10 (5.3%)	33 (17.6%)	4 (2.2%)	9 (5.0%)	12 (6.6%)			
**VPI** (mean ± SD)(0 ~ 1) ^**§**^	0.19 ± 0.16^a^	0.14 ± 0.13^b^	0.15 ± 0.14^b^	0.19 ± 0.15^a^	0.16 ± 0.16^b^	0.22 ± 0.17^a^	<0.001	<0.001	0.025
VPI > 0 (*n*,%)	157 (83.5%)	138 (73.4%)	141 (75.0%)	149 (82.3%)	130 (71.8%)	151 (73.4%)			
VPI > 0.5 (*n*,%)	7 (3.7%)	1 (0.5%)	0 (0%)	4 (2.2%)	4 (2.2%)	9 (5%)			
**BOP%** (mean + SD)(0 ~ 100%) ^**§**^	57 ± 27^a^	46 ± 30^b^	35 ± 29^c^	58 ± 27^a^	52 ± 29^b^	46 ± 26^c^	< 0.001	0.001	0.014
BOP> 0 (*n*,%)	181 (96.3%)	166 (88.3%)	151 (75.0%)	168 (92.8%)	164 (90.6%)	151 (83.4%)			
BOP> 50% (*n*,%)	103 (54.8%)	71 (37.8%)	48 (25.5%)	101 (55.8%)	84 (46.4%)	64 (35.4%)			
**NPoc** (mean + SD)(0 ~ 6) ^¶^	0.22 ± 0.25^a^	0.29 ± 0.19^b^	0.08 ± 0.16^c^	0.21 ± 0.25^a^	0.30 ± 0.16^b^	0.09 ± 0.17^c^	< 0.001	0.293	0.402
0-3 mm (*n*,%)	165 (87.8%)	162 (86.2%)	179 (95.2%)	165 (91.2%)	143 (79.0%)	172 (95.0%)			
> 3 mm (*n*,%)	23 (12.2%)	26 (13.8%)	9 (4.8%)	16 (8.8%)	38 (21.0%)	9 (5.0%)			
**NLoA** (mean + SD)(0 ~ 6) ^¶^	0.23 ± 0.24^a^	0.22 ± 0.18^a^	0.12 ± 0.16^b^	0.21 ± 0.15^a^	0.21 ± 0.16^a^	0.10 ± 0.17^b^	< 0.001	0.445	0.352
0-3 mm (*n*,%)	167 (88.8%)	168 (89.4%)	179 (95.2%)	165 (91.2%)	154 (85.1%)	172 (95.0%)			
> 3 mm (*n*,%)	21 (11.2%)	20 (10.6%)	9 (4.8%)	16 (8.8%)	27 (14.9%)	9 (5.0%)			

*VPI* proportion of tooth surface with visible dental plaque

*BOP%* percentage of sites with gingival bleeding

*NPoc* number of sextants with periodontal pocket > 3 mm

*NLoA* number of sextants with loss of attachment > 3 mm

Each subscript letter denotes a subset of means that do not differ significantly from each other at the 0.05 level

^**§**^ *p*-value obtained using mixed-effect ANOVA

^¶^ *p*-value obtained using generalized linear mixed model

At one year postpartum, more participants had healthy periodontal condition than at baseline (McNemar’s test, *p* < 0.001). Meanwhile, the test group had a higher proportion of individuals with healthy periodontal status than the control group one year postpartum (Chi-square test, *p* = 0.001) (Table 3).

At T_0_, around 3% of the participants had visible plaque on more than 50% of the tooth surfaces. This proportion decreased after the intervention in the test group (3.7% at T_0_; 0.5% at T_1_; and 0% at T_2_). In contrast, the proportion of participants in the control group with this condition increased at T_2_ (2.2% at T_0_; 2.2% at T_1_; 5% at T_2_). The mean VPI score in the test group was 0.19 at T_0_, which decreased to 0.14 at T_1_ and remained at this low level at T_2_. In the control group, the mean VPI score slightly reduced from 0.19 at baseline to 0.16 at T_1_, but increased to 0.22 at T_2_. A main effect of time and intervention, as well as an interaction between time and intervention, was detected (all *p* < 0.05), indicating that the intervention effect differed between T_1_ and T_2_. The between-group difference in the change of VPI score was statistically significant (*p* = 0.025). Pairwise comparisons were carried out with Bonferroni adjustment. From T_0_ to T_1_, the decrease in mean VPI score was statistically significant in the test group (*p* < 0.001) but not in the control group (*p* > 0.05). At T_2_ (12 months postpartum), the mean VPI score of the test group was similar to the corresponding T_1_ values and remained significantly better than baseline (*p* = 0.01). However, the mean VPI score was significantly higher at T_2_ compared to T_1_ in the control group.

Throughout the study, the proportion of pregnant women with a severe condition of gingival bleeding (> 50% examined sites) decreased in both study groups. However, the prevalence of severe gingival bleeding was lower in the test group than in the control group at T_2_ (*p* < 0.01). The trend of change in BOP% was similar to that of VPI. In the test group, the mean BOP% was 57% at T_0_ and decreased to 46 and 35% at T_1_ and T_2_, respectively. The mean BOP% in the control group only slightly reduced from 58% at baseline to 52 and 46% at T_1_ and T_2_. A main effect of time and intervention, as well as an interaction between time and intervention, was detected (all *p* < 0.05), indicating that the intervention effect differed between T_1_ and T_2_. The test group showed a more significant decrease in BOP than the control group did.

Compared with T_0_, more pregnant women had a Poc at T_1_ (*p* < 0.01), and fewer participants had a Poc at T_2_ (*p* < 0.001). No significant difference was found between the two study groups at all three time points (*p* > 0.05). In both groups, the mean NPoc increased from T_0_ (around 0.2) to T_1_ (about 0.3) and then decreased at T_2_ (about 0.1). There were significant changes over time in both groups (*p* < 0.001). There was no significant intervention-time interaction, such that the intervention effect did not differ between T_1_ and T_2_ (*p* > 0.05). The finding for the NLoA was similar. There was a statistically significant change over time (*p* < 0.001). The mean NLoA at T_2_ was significantly lower than at T_0_ and T_1_. There was no significant difference between the two groups over time (*p* > 0.05), and no interaction (*p* > 0.05) was observed.

## Discussion

The findings of this clinical trial show that the family-centered behavioral and educational counselling approach with reinforcement is more effective in improving the oral hygiene and gingival health status of pregnant women than traditional OHE by distributing pamphlets alone. Pregnancy does not necessarily lead to more severe gingivitis or periodontitis if pregnant women can be empowered to maintain good oral hygiene.

The favorable outcomes resulting from the behavioral and education counselling approach may be due to several reasons. First, even though the intervention was not developed based on a psychological theory or model, it shared some essential components of the Health Belief Model for promoting health [25]. After the behavioral and education counselling, the pregnant women were aware that they were more susceptible to periodontal disease during pregnancy (perceived susceptibility); realized that periodontal disease could have potentially severe consequences, such as preterm delivery (perceived severity); and believed that good oral hygiene practice would produce positive outcomes, such as reducing the vertical transmission of oral cariogenic bacteria to children (perceived benefits). Furthermore, the communication with the oral health educator during the reinforcements provided an opportunity to solve the problems they encountered (perceived barriers). Generally, theory-based interventions are proven to be more successful in achieving stable and permanent behavioral changes than non-theory-based interventions [26–28]. Second, there was a detailed demonstration of oral hygiene practice adopting the *“tell-show-do”* approach in this study. Training on the correct toothbrushing method was delivered to the participants and focused on areas that are particularly relevant to pregnant women, such as cleaning the gingival margin of the teeth, handling nausea/vomiting when brushing teeth, and using dental floss to clean proximal surfaces. All these procedures and the feedback provided to the participants were useful to improve their capabilities to perform proper oral hygiene measures. Third, this study targeted first-time expectant mothers at their early pregnancy stage. Pregnancy, especially the first one, is a significant transition for a woman that provides an opportunity to “change” (e.g., change of nutrition-related behavior because the woman has concerns about the health of herself and her fetus) [29]. Similarly, first pregnancy may be an excellent opportunity to elicit positive changes in oral health-related behavior. Findings of a previous study suggest that pregnant women in their first trimester are more willing to participate in research projects and to receive prenatal or other health care [30]. Fourth, the intervention was family centered in that the husbands received the OHE together with the pregnant women, with reinforcement at different time points. Findings of a systematic review support that health interventions involving men can increase care seeking, improve home care practices and couple relationships, and promote women’s health [31].

This study sheds light on the impact of an oral health promotion program targeting pregnant women in a primary care setting, which has been sparse in the literature. However, this study had several limitations. First, although the participants were recruited from public prenatal care centers in different districts of Hong Kong, their socioeconomic status was higher than the average of the general population in Hong Kong. This could have led to a better response to oral health promotion. More research on interventions delivered to other populations (e.g., low-income people) is required to confirm if these findings can be generalized. Second, there was a methodological limitation in adopting the indices and the partial periodontal examination recommended by the WHO [22]. Understandably, partial mouth recordings are quicker to undertake than full mouth recordings. Although this approach may result in underestimation of the disease [32], it was essential and helpful in this study because our participants were pregnant women and the data collection was carried out during their prenatal care with limited time. This measurement approach has been recognized as being well suited for identifying individuals who are (and who continue to be) periodontally healthy [33, 34]. Thus, we can conclude that individualized behavioral and educational counselling produces promising results for maintaining good periodontal health among pregnant women.

Compared with that of the local population [35], the oral hygiene status of the participants in this study was better. Although 82.5% of them had visible plaque on the examined tooth surfaces, only about 3% of the participants had visible plaque on half or more of the surfaces. There is a potential “ceiling effect” (i.e., the reasonably good oral hygiene at baseline would make further improvement difficult). Indeed, greater efficacy could be expected if the intervention were delivered to people with poorer oral hygiene.

This study demonstrates that behavioral and educational counselling could be an effective way to maintain good periodontal health in pregnant women. A pregnant woman could receive adequate information and oral hygiene instructions from dental or non-dental health professionals (e.g., her obstetrician, midwives, nurses) and be made aware of how to improve her oral health. Psychological theory-based oral health promotion is suggested and could be incorporated into routine dental care or integrated into prenatal care. Furthermore, such an intervention should be implemented at an early stage of pregnancy, so that reinforcement sessions can be scheduled if necessary.

## Conclusion

Providing family-centered, behavioral, and educational counselling to pregnant women at an early stage of pregnancy, and with reinforcement, can improve their oral hygiene and reduce gingival inflammation. The effect can be sustained over a more extended period than that achieved by distributing oral health leaflets alone.

## Data Availability

The full trial protocol accessed, datasets used and/or analyzed during the current study are available from the corresponding author on reasonable request.

## Abbreviations

BOP: Bleeding on probing
BOP%: Percentage of sites with BOP
ECC: Early childhood caries
LoA: Loss of clinical attachment
NLoA: Number of sextants with LoA > 3 mm
Poc: Periodontal pocket
NPoc: Number of sextants with Poc > 3 mm
OHE: Oral health education
VPI: Visible Plaque Index
WHO: World Health Organization
